# Structures and functions of the normal and injured human olfactory epithelium

**DOI:** 10.3389/fncir.2024.1406218

**Published:** 2024-06-06

**Authors:** Shu Kikuta, Shin Nagayama, Sanae Hasegawa-Ishii

**Affiliations:** ^1^Department of Otolaryngology-Head and Neck Surgery, Faculty of Medicine, Nihon University, Tokyo, Japan; ^2^Department of Neurobiology and Anatomy, McGovern Medical School at The University of Texas Health Science Center at Houston, Houston, TX, United States; ^3^Pathology Research Team, Kyorin University, Tokyo, Japan

**Keywords:** olfactory epithelium, olfactory dysfunction, respiratory metaplasia, chronic rhinosinusitis, viral infection

## Abstract

The olfactory epithelium (OE) is directly exposed to environmental agents entering the nasal cavity, leaving OSNs prone to injury and degeneration. The causes of olfactory dysfunction are diverse and include head trauma, neurodegenerative diseases, and aging, but the main causes are chronic rhinosinusitis (CRS) and viral infections. In CRS and viral infections, reduced airflow due to local inflammation, inflammatory cytokine production, release of degranulated proteins from eosinophils, and cell injury lead to decreased olfactory function. It is well known that injury-induced loss of mature OSNs in the adult OE causes massive regeneration of new OSNs within a few months through the proliferation and differentiation of progenitor basal cells that are subsequently incorporated into olfactory neural circuits. Although normal olfactory function returns after injury in most cases, prolonged olfactory impairment and lack of improvement in olfactory function in some cases poses a major clinical problem. Persistent inflammation or severe injury in the OE results in morphological changes in the OE and respiratory epithelium and decreases the number of mature OSNs, resulting in irreversible loss of olfactory function. In this review, we discuss the histological structure and distribution of the human OE, and the pathogenesis of olfactory dysfunction associated with CRS and viral infection.

## Introduction

The sense of smell is extensively used in everyday life, from the perception of danger signals, such as smoke and noxious gases to the detection of spoiled food and the psychosocial effects of food (Croy et al., 2014; Rebholz et al., 2020).

The etiology of olfactory dysfunction varies widely and includes chronic rhinosinusitis (CRS), upper respiratory tract viral infection, head trauma, allergic rhinitis, and aging. However, among these, CRS and viral infection account for about 60% of all cases (Seiden, 2004; Rombaux et al., 2016). In CRS and viral infections, olfactory perception is reduced due to decreased airflow caused by mucosal swelling and polyp formation, as well as by injury to olfactory sensory neurons (OSNs) by pathogens such as viruses, bacteria, eosinophil granule products, and inflammatory cytokines (Kern, 2000; Seiden, 2004; Wrobel and Leopold, 2004).

Different types of olfactory epithelium (OE) injury and OE regeneration after injury have been reported (Imamura and Hasegawa-Ishii, 2016). Irrespective of the type of OE injury, tissue regeneration is usually complete within 1–2 months after injury (Kikuta et al., 2015; Imamura and Hasegawa-Ishii, 2016). However, some patients with CRS experience a decreased sense of smell despite having an open olfactory cleft and normal nasal airflow, or show no improvement in olfactory function despite polyp removal (Apter et al., 1992; Kikuta et al., 2016). Similarly, olfactory dysfunction caused by viral infections takes time to improve and may persist for months to a year and more (Liu et al., 2023). Therefore, understanding the anatomical or histological characteristics of the human OE, as well as the histological changes and pathophysiology after injury, is essential for developing appropriate treatment strategies for prolonged olfactory dysfunction.

This review describes the histological features of the human OE and discusses the pathogenesis of olfactory dysfunction associated with CRS and viral infections, as well as persistent olfactory dysfunction.

## Odor reception in humans

The ciliary membranes of the OSN contain olfactory receptors (ORs), which are responsible for odor detection. ORs are members of the G-protein-coupled seven-transmembrane receptor family and constitute the largest gene family (Buck and Axel, 1991; Buck, 2000) in the human genome with nearly 400 OR-coding genes (Zozulya et al., 2001; Young et al., 2002; Zhang and Firestein, 2002; Godfrey et al., 2004; Malnic et al., 2004). The OE is divided into zones I, II, III, and IV (from the dorsomedial to ventral region), which contain densely packed OSNs.

In many vertebrates including humans, OR genes are classified into two classes, class I and class II, based on differences in their amino acid sequences (Glusman et al., 2001; Imai et al., 2010). OSNs expressing class I genes (class I OSNs) are distributed within zone 1, corresponding to the dorsomedial region of the OE, while OSNs expressing class II genes (class II OSNs) are widely distributed in zones II-IV (Mori and Sakano, 2011). The presence of a zone structure in the OE has not been confirmed in human, but in the macaque, a higher primate phylogenetically related to humans, OSNs expressing specific ORs are scattered throughout the OE but are restricted to specific zones, suggesting the presence of a zone structure (Ressler et al., 1993; Horowitz et al., 2014; Mori and Sakano, 2021).

## Cellular composition of the human OE

**FIGURE 1:**
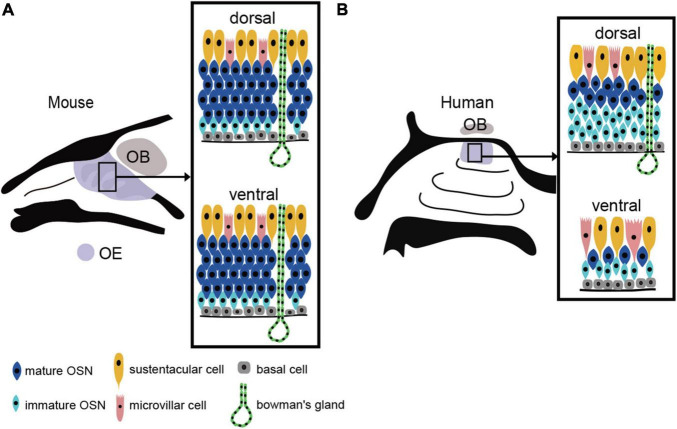
OE structure and the distribution of the OE in mouse and human. **(A)** Uniform distribution of the mouse OE. In mouse, OSNs are arranged in a laminar pattern and are evenly distributed in both the dorsal and ventral regions of the OE. OSN, olfactory sensory neuron; OE, olfactory epithelium; OB, olfactory bulb; BC, basal cell; MVC, microvillar cell; SC, sustentacular cell. **(B)** Heterogeneous distribution of the human OE. The human OE lacks a well-defined stratified structure and is generally more sparsely distributed. It also has a lower density of OSNs than mouse. The dorsal surface of the OE contains more mature OSNs, while the ventral surface contains fewer mature OSNs.

The human OE lacks the distinct laminar structure observed in the mouse OE. The OSN density is very low and the OSNs are sparsely distributed (Omura et al., 2022). Furthermore, OSN density is not uniform; mature OSNs are abundant and present at a relatively high density near the cribriform plate (dorsal to the nasal cavity), but their density gradually decreases with distance from the cribriform plate (Figure 1).

The human OE is composed of five cell types: immature or mature OSNs, sustentacular cells (SCs), microvillar cells (MVCs), tubular cells of Bowman’s glands, and basal cells (BCs) (Moran et al., 1982; Morrison and Costanzo, 1990; Féron et al., 1998; Kalinke et al., 2011).

OSNs are bipolar neurons that extend one dendrite on the surface of the OE to the mucus layer and project one unmyelinated axon to the olfactory bulb (OB) (Yee et al., 2010). Individual OSN dendrites have olfactory vesicles at their tips and are attached to 10–15 non-motile, elongated cilia. The axons of OSNs cross the basal membrane and merge to form non-myelinated nerve bundles called fascicles (Jafek, 1983; Morrison and Costanzo, 1990).

SCs are tall cells with a nucleus on the apical side that extend their projections from the surface of the epithelium to the basal layer (Jafek, 1983; Morrison and Costanzo, 1990). Two or more neighboring SCs wrap around the dendrites of OSNs, structurally and electrically isolating the OSN (Bryche et al., 2020). They are also involved in supplying glucose to OSNs and maintaining ion balance within the OE (Vogalis et al., 2005; Lemons et al., 2017; O’leary et al., 2019; Ualiyeva et al., 2020). Furthermore, SCs defend the OE by phagocytosing and detoxifying olfactory toxins using metabolic enzymes such as cytochrome P450 and glutathione S-transferase. They also contribute to local immunity by producing inflammatory cytokines when local inflammation persists (Jafek, 1983).

MVCs are non-neuronal cells with rigid microvilli and some express TRPM5. TRPM5-postive cells express choline acetyltransferase and the vesicular acetylcholine transporter (Ogura et al., 2011; Saunders et al., 2014) and stimulate SCs by releasing acetylcholine, which protects the OE by promoting the metabolism and removal of olfactory toxicants (Ogura et al., 2011; Genovese and Tizzano, 2018).

Bowman’s glands are spaced across the basal membrane at regular intervals and are responsible for the production and secretion of mucus, which covers the luminal surface of the OE.

BCs are spherical stem cells that differentiate into OSNs (Morrison and Costanzo, 1990). Unlike in mice, there is no distinction between horizontal basal cells (HBCs) and globose basal cells (GBCs) in human (Graziadei and Graziadei, 1979; Holbrook et al., 2011), but morphologically, human BCs resemble GBCs in mice (Hahn et al., 2005).

## Distribution of the human OE

In human, the proportion of the nasal cavity occupied by the OE is markedly lower than that in rodents; in rats, the OE accounts for about 50% of the nasal cavity, whereas in human, it occupies about 3% (Gross et al., 1982).

The human nasal cavity consists of three nasal concha (superior, middle, and inferior). The OE is localized in the superior nasal concha, particularly in a limited area corresponding to its upper anterior two-thirds (Omura et al., 2022). In mice, the respiratory epithelia (RE) and OE are clearly distinguishable and are not histologically intermingled. However, in human, the boundary between RE and OE is unclear and is characterized by patchy areas of mixed RE and OE (Nakashima et al., 1984; Morrison and Costanzo, 1990; Omura et al., 2022). Areas of OE degeneration and respiratory epithelialization increase with age (Nakashima et al., 1984; Paik et al., 1992), but do not necessarily correlate with loss of olfactory function, because OE degeneration and OSN reduction are also observed in adults with normal olfactory function (Nakashima et al., 1984; Omura et al., 2022).

## OE injury is associated with CRS

CRS is defined as a chronic inflammatory disease of the sinus mucosa that persists for more than 3 months (Fokkens et al., 2020), and is the most frequent etiology of olfactory dysfunction (Rombaux et al., 2016). Approximately 60–80% of CRS patients experience a decreased sense of smell (Banglawala et al., 2014). CRS phenotypes are classified into two types: CRS with nasal polyps (CRSwNP) and CRS without nasal polyps (CRSsNP) (Fokkens et al., 2020). CRSwNP causes a high rate of olfactory dysfunction and is associated with eosinophil-driven inflammation, eosinophilic cationic proteins (ECPs), and injury to OSNs by inflammatory cytokines released from eosinophils (Epstein et al., 2008; Li et al., 2010; Acharya and Ackerman, 2014; Yan X. et al., 2020).

In animal models of CRS, in addition to the release of inflammatory cytokines (such as TNF-a and interferon-c) from SCs and OSN cell death, BC proliferation and differentiation are arrested, resulting in neuroepithelium remodeling and the replacement of neuroepithelium with RE (Jafek et al., 2002; Yee et al., 2009; Lane et al., 2010; Goncalves and Goldstein, 2016; Choi and Goldstein, 2018; Marin et al., 2022). Furthermore, prolonged inflammation increases c-Jun N-terminal kinase activity, a promoter of apoptosis, within the OSN and local eosinophil infiltration (Victores et al., 2018). Intranasal administration of ECP to mouse OEs for 2 weeks results in OSN apoptosis and thinning of OEs, similar to previous observations in human (Kikuta et al., 2021). Interestingly, ECP induces apoptosis not only in OSNs but also in some BCs. In fact, histological analysis of human OEs frequently showed massive infiltration of inflammatory cells, such as lymphocytes, macrophages, and eosinophils, and reduced numbers of OSNs and squamous metaplasia (Kern, 2000; Rombaux et al., 2016; Wu et al., 2020; Marin et al., 2022). Consistent with the location of direct injury to the OE, axonal bundles may fail to extend from the OE beyond the basal membrane and are observed within the OE as tangles of nerve fibers (Holbrook et al., 2005). Indeed, the degree of the OE inflammation and eosinophil infiltration correlates closely with reduced olfaction in CRS patients (Soler et al., 2009; Kashiwagi et al., 2019). Furthermore, persistent inflammation leads to increased mucus secretion from Bowman’s glands, and disruption of the balance of potassium and sodium ion concentrations in the mucus reduces olfactory reception (Selvaraj et al., 2012; Rombaux et al., 2016).

## OE injury following viral infection

Post-viral olfactory dysfunction (PVOD) is the second most common type of olfactory dysfunction and accounts for about 30% of patients with olfactory dysfunction (Seiden, 2004). Many viruses have been reported to infect OSNs, including influenza A virus (Van Riel et al., 2014), herpes virus (Esiri, 1982), paramyxo virus (Van Riel et al., 2015), parainfluenza virus (Van Riel et al., 2015), adeno virus (Yamada et al., 2009), and Japanese encephalitis virus (Yamada et al., 2009), based on the analysis of samples and tissues obtained from experimentally inoculated animals. These viruses utilize various receptors such as the sialic acid (e.g., influenza virus, parainfluenza virus, and adenovirus) (Connor et al., 1994; Villar and Barroso, 2006) and heparan sulfate receptors (e.g., herpes virus and Japanese encephalitis virus) (Eisenberg et al., 2012; Perera-Lecoin et al., 2013) to enter the OSN. Although these receptors are similarly expressed in human OSNs, it is still unclear whether these viruses directly enter human OSNs. Seasonal influenza virus A (H3N2), pandemic influenza virus A (H1N1), and highly pathogenic avian influenza virus A (H5N1) have been shown to attach to the apical side of human OSNs, and it is suggested that these viruses can infect human OSNs (Van Riel et al., 2015). Biopsies of the olfactory mucosa of patients with PVOD show OE degeneration and morphological changes in the RE (Seiden, 2004). In addition, in the mucosal intrinsic layer below the basal membrane, OSN axons are prominently replaced by collagen fibers (Holbrook et al., 2005).

Olfactory tests employing combinations of odors with different chemical structures has been used to characterize OE damage in patients with PVOD (Kikuta et al., 2023). Olfactory stimulation with β-phenylethyl alcohol, γ-undecalactone, and isovaleric acid, known as the T&T olfactometer test in Japan, allows PVOD patients to discriminate between different odors, while odor stimulation with prosultiamine, known as the intravenous olfactory (IVO) test in Japan, does not allow odor discrimination. The former group of odors activates both class I and class II OSNs, while the latter odor primarily activates class I OSNs (Takahashi et al., 2004; Igarashi and Mori, 2005; Mori and Sakano, 2011; Kikuta et al., 2023). Thus, it has been suggested that virus-induced OE injury may occur heterogeneously in a cell type-dependent manner, with preferential injury to class I OSNs (Kikuta et al., 2023).

Coronavirus disease 2019 (COVID-19) is caused by SARS-CoV-2, but the mechanism of infection differs from that of other viruses that cause the common cold (Belouzard et al., 2012). In addition to the angiotensin-converting enzyme 2 (ACE2) receptor, transmembrane protease serine 2 (TMPRSS2) activity is required for SARS-CoV-2 infection (Hoffmann et al., 2020). Co-expression of ACE2 and TMPRSS2 has been observed only in SCs, Bowman’s glands, MVs, and BCs, but not in OSNs (Cooper et al., 2020). In golden hamsters, SARS-CoV-2 infects intestinal cells but not OSNs (Bryche et al., 2020). OE samples from COVID-19 patients have been found to contain coronavirus antigens in cells within the OE, and although the type of infected cell has not been identified, their shape and antigen localization suggest that the virus targets non-OSN cells (Cantuti-Castelvetri et al., 2020). However, infection of non-OSN cells increases inflammatory cytokines in the human OE (Torabi et al., 2020), and in experiments using golden hamsters, shedding of OSN cilia was observed histologically (Bryche et al., 2020).

## Discussion

The human OE lacks a regular laminar structure, and a mixture of the RE within the OE is observed even in people with a normal sense of smell. With respect to OSN density, the dorsal OE has a higher density of mature olfactory OSNs than the ventral OE (Figure 1). The areas of low OSN density in the ventral OE coincide with areas of high airflow, suggesting that airflow is a chronic mechanical stimulus affecting the OE and that the epithelium in this area of the OE may be degenerative. Since mice show no such differences in OSN density, it is possible that the human OE is especially susceptible to injury from airflow stimulation.

**FIGURE 2:**
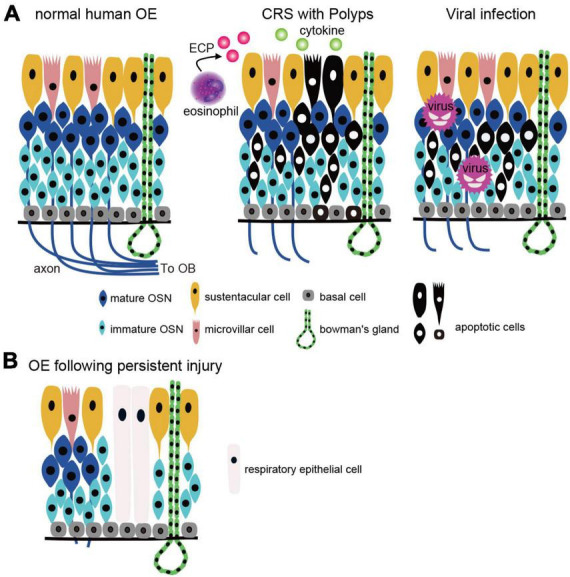
OE injury caused by CRS and viral infection. **(A)** Two types of the OE injury. Left, normal human OE: OSN axons extend to the OB and form synapses with projection neurons in the OB. Middle, CRS with polyps: OE is injured by inflammatory cytokines and eosinophilic cationic proteins from eosinophils, resulting in apoptotic cell death. Right, viral infection: viruses (except COVID-19) infect OSNs directly and cause apoptotic cell death. CRS, chronic rhinosinusitis; OSN, olfactory sensory neuron; OE, olfactory epithelium; OB, olfactory bulb; BC, basal cell; MVC, microvillar cell; SC, sustentacular cell; ECP, eosinophilic cationic protein. **(B)** Histological changes after OE injury. Persistent inflammation and basal cell damage in the OE can inhibit axonal elongation of newly generated OSNs and/or result in a transition from olfactory to respiratory epithelium, leading to prolonged olfactory dysfunction.

The olfactory loss associated with CRS is caused by mechanical obstruction of the olfactory cleft by polyps in the nasal cavity and/or edematous changes in the nasal mucosa, resulting in reduced airflow to the OE (Kern, 2000; Banglawala et al., 2014; Rosenfeld et al., 2015; Gudis and Soler, 2016). In addition, direct injury to the OE by inflammatory cytokines and degranulation proteins from eosinophils also reduce olfactory function (Aiba and Nakai, 1991; Doty and Mishra, 2001; Lane et al., 2010). In mouse experiments, repetitive injury, such as chronic inflammation and aging have been reported to activate HBCs, which depletes their potential to produce OSNs (Håglin et al., 2020). Eventually, HBCs produce respiratory epithelial cells instead of OSNs by altering retinoic acid metabolism and are involved in respiratory transformation (Håglin et al., 2020). Thus, severe inflammation and BC damage to the OE leads to prolonged olfactory dysfunction by reducing the number of functional OSNs and promoting respiratory transformation (Figure 2). However, since respiratory transformation is observed even in adults with normal olfactory function, it is unclear to what extent OE degeneration must progress before olfactory loss becomes apparent. Rats can detect food odors even after more than 90% of the olfactory mucosa has degenerated (Youngentob et al., 1997), suggesting that the peripheral olfactory system has significant reserve capacity. If this is also the case in humans, patients who are aware of their decreased sense of smell may be in the final stages of extensive OE degeneration.

It is unclear whether differences exist in olfactory dysfunction between aging and CRS, but histological changes in OB may produce differences in olfactory function. Human studies have reported that OB volume, the thickness of the glomerular layer, and the number of mitral cells and glomeruli decrease with age (Bhatnagar et al., 1987; Meisami et al., 1998; Yousem et al., 1998). Furthermore, cell division in the mouse subventricular zone decreases with age, but granule cell density in the OB increases with age (Enwere et al., 2004; Richard et al., 2010), suggesting that granule cell turnover in the OB is reduced and granule cells live longer in aging animals (Sui et al., 2012). Decreased turnover of granule cells with aging may reduce the likelihood of neural circuit reorganization. On the other hand, persistent nasal inflammation in mice treated with lipopolysaccharide causes marked atrophy of the OB and more intense damage in tufted cells than in mitral cells (Hasegawa-Ishii et al., 2019, 2020). Furthermore, peripheral immune cells have been shown to transiently infiltrate the olfactory nerve layer, the glomerular layer, and the external plexiform layer, suggesting that chronic inflammation, including CRS, may also induce histological changes in the OB (Asano et al., 2022). A detailed study of the relationship between olfactory function and histological changes in the OB may reveal differences in olfactory dysfunction between aging and chronic inflammation.

In contrast to olfactory loss caused by reduced airflow, which can be improved by surgical treatment, no established treatment currently exists for OE injury. Therefore, to develop a treatment strategy, it is important to determine whether olfactory dysfunction is due solely to reduced airflow or to concomitant OE damage. The IVO test measures the time (defined as latency) and duration of odor perception after intravenous administration of prosultiamine and is widely used in clinical practice. Reduced airflow does not prolong onset latency in the IVO test, but OE injury does, and prolonged latency correlates with a reduction in the number of mature OSNs (Kikuta et al., 2016). Accordingly, OE injury is likely in cases with prolonged latency, and it is estimated that approximately 60% of CRS cases are complicated by OE injury (Kikuta et al., 2016).

The main mechanism of PVOD is decreased airflow caused by swelling of the olfactory cleft mucosa by local inflammation, increased mucus production, and changes in mucus composition (Akerlund et al., 1995; Schlosser et al., 2016; Victores et al., 2018; Cooper et al., 2020). Thus, in most cases, olfactory function recovers with the disappearance of nasal symptoms (Hummel et al., 1998a,b; Zhao et al., 2014), but in some patients, olfactory loss may persist for more than a year. This is presumably due to the OE damage caused by viral infection or the local immune response (Duncan and Seiden, 1995; Welge-Lüssen and Wolfensberger, 2006; Cavazzana et al., 2018). Viruses that invade OSNs can be transported to the olfactory bulb (OB) via OSN axons. However, OSN apoptosis, a defense mechanism against OE damage, can prevent this propagation (Mori et al., 2002, 2004; Kanaya et al., 2014). When mice are infected intranasally with influenza H3N1 virus, apoptosis of the infected OSNs inhibits the spread of the viruses. Conversely, infection with herpes viruses does not induce OSN apoptosis and the viruses can spread to the OB (Mori et al., 2002). Thus, OSN apoptosis may act positively by preventing the entry of viruses into the central nervous system via the OSN, but may also act negatively by promoting olfactory dysfunction.

Olfactory dysfunction caused by COVID-19 is less severe than that caused by other common cold viruses, with olfaction restored in about 70% of cases within 2 weeks after the onset of initial symptoms (Lechien et al., 2020; Yan C. H. et al., 2020). The fact that COVID-19 infects SCs, MVs, and BCs, but not OSNs may be one factor contributing to the favorable prognosis of olfactory dysfunction in COVID-19 patients (Belouzard et al., 2012; Cooper et al., 2020).

Regardless of the type of virus, severe injury to OSNs and other components of the OE can result in incomplete regeneration, and similar to the histopathology of CRS, degeneration and morphological changes in the OE are observed (Seiden, 2004; Figure 2). The histological changes that occur during the regenerative process may be one of the factors contributing to the prolongation of olfactory symptoms.

Since a variety of immune cells are known to be involved in inflammatory responses in the OE, research into the types and activities of the immune cells involved will be required to elucidate the mechanisms before efficacious treatments for olfactory dysfunction can be developed. However, various therapeutic interventions with variable efficacy are available, including steroid administration, which has anti-inflammatory effects, for the treatment of olfactory dysfunction caused by CRS (Rudmik et al., 2013; Chang and Glezer, 2018). Biological therapies such as anti-IgE monoclonal antibody, IL-4 receptor alpha subunit antagonist, and anti-IL-5 are promising treatments for nasal polyps and could significantly improve olfaction (Gevaert et al., 2013; Bachert et al., 2016, 2017). In addition, localized intranasal administration of insulin in mice has been reported to suppress OSN apoptosis and promote OE regeneration, suggesting that insulin could have potential as a novel therapeutic agent (Kikuta et al., 2021; Kuboki et al., 2021). In human, insulin nasal spray has also been reported to be effective against COVID-19-induced olfactory dysfunction (Cherobin et al., 2023). Understanding the histological architecture of the human OE and the pathophysiology of each disease will be fundamental in establishing new therapies for controlling inflammation and preventing irreversible OE damage.

## Author contribution

SK: Conceptualization, Data curation, Funding acquisition, Project administration, Validation, Writing–original draft, Writing–review and editing, Methodology. SN: Writing–review and editing. SH-I: Writing–review and editing.

## References

[B1] Acharya K. R. Ackerman S. J. (2014). Eosinophil granule proteins: Form and function. *J. Biol. Chem.* 289 17406–17415. 10.1074/jbc.R113.546218 24802755 PMC4067173

[B2] Aiba T. Nakai Y. (1991). Influence of experimental rhino-sinusitis on olfactory epithelium. *Acta Otolaryngol. Suppl.* 486 184–192. 10.3109/00016489109134995 1842866

[B3] Akerlund A. Bende M. Murphy C. (1995). Olfactory threshold and nasal mucosal changes in experimentally induced common cold. *Acta Otolaryngol.* 115 88–92. 10.3109/00016489509133353 7762392

[B4] Apter A. J. Mott A. E. Cain W. S. Spiro J. D. Barwick M. C. (1992). Olfactory loss and allergic rhinitis. *J. Allergy Clin. Immunol.* 90 670–680. 10.1016/0091-6749(92)90141-N 1401646

[B5] Asano H. Hasegawa-Ishii S. Arae K. Obara A. Laumet G. Dantzer R. (2022). Infiltration of peripheral immune cells into the olfactory bulb in a mouse model of acute nasal inflammation. *J. Neuroimmunol.* 368:577897. 10.1016/j.jneuroim.2022.577897 35661951 PMC9903215

[B6] Bachert C. Mannent L. Naclerio R. M. Mullol J. Ferguson B. J. Gevaert P. (2016). Effect of subcutaneous dupilumab on nasal polyp burden in patients with chronic sinusitis and nasal polyposis: A randomized clinical trial. *JAMA* 315 469–479. 10.1001/jama.2015.19330 26836729

[B7] Bachert C. Sousa A. R. Lund V. J. Scadding G. K. Gevaert P. Nasser S. (2017). Reduced need for surgery in severe nasal polyposis with mepolizumab: Randomized trial. *J. Allergy Clin. Immunol.* 140:1024–1031.e1014. 10.1016/j.jaci.2017.05.044 28687232

[B8] Banglawala S. M. Oyer S. L. Lohia S. Psaltis A. J. Soler Z. M. Schlosser R. J. (2014). Olfactory outcomes in chronic rhinosinusitis with nasal polyposis after medical treatments: A systematic review and meta-analysis. *Int. Forum Allergy Rhinol.* 4 986–994. 10.1002/alr.21373 25400017

[B9] Belouzard S. Millet J. K. Licitra B. N. Whittaker G. R. (2012). Mechanisms of coronavirus cell entry mediated by the viral spike protein. *Viruses* 4 1011–1033. 10.3390/v4061011 22816037 PMC3397359

[B10] Bhatnagar K. P. Kennedy R. C. Baron G. Greenberg R. A. (1987). Number of mitral cells and the bulb volume in the aging human olfactory bulb: A quantitative morphological study. *Anat. Rec.* 218 73–87. 10.1002/ar.1092180112 3605663

[B11] Bryche B. St Albin A. Murri S. Lacôte S. Pulido C. Ar Gouilh M. (2020). Massive transient damage of the olfactory epithelium associated with infection of sustentacular cells by SARS-CoV-2 in golden Syrian hamsters. *Brain Behav. Immun.* 89 579–586. 10.1016/j.bbi.2020.06.032 32629042 PMC7332942

[B12] Buck L. B. (2000). The molecular architecture of odor and pheromone sensing in mammals. *Cell* 100 611–618. 10.1016/S0092-8674(00)80698-4 10761927

[B13] Buck L. Axel R. (1991). A novel multigene family may encode odorant receptors: A molecular basis for odor recognition. *Cell* 65 175–187. 10.1016/0092-8674(91)90418-X 1840504

[B14] Cantuti-Castelvetri L. Ojha R. Pedro L. D. Djannatian M. Franz J. Kuivanen S. (2020). Neuropilin-1 facilitates SARS-CoV-2 cell entry and infectivity. *Science* 370 856–860. 10.1126/science.abd2985 33082293 PMC7857391

[B15] Cavazzana A. Larsson M. Münch M. Hähner A. Hummel T. (2018). Postinfectious olfactory loss: A retrospective study on 791 patients. *Laryngoscope* 128 10–15. 10.1002/lary.26606 28556265

[B16] Chang S. Y. Glezer I. (2018). The balance between efficient anti-inflammatory treatment and neuronal regeneration in the olfactory epithelium. *Neural Regen. Res.* 13 1711–1714. 10.4103/1673-5374.238605 30136681 PMC6128054

[B17] Cherobin G. B. Guimarães R. E. S. De Paula Gomes M. C. Vasconcelos L. O. G. De Abreu L. N. (2023). Intranasal insulin for the treatment of persistent post-COVID-19 olfactory dysfunction. *Otolaryngol. Head Neck Surg.* 169 719–724. 10.1002/ohn.352 37078341

[B18] Choi R. Goldstein B. J. (2018). Olfactory epithelium: Cells, clinical disorders, and insights from an adult stem cell niche. *Laryngosc. Invest. Otolaryngol.* 3 35–42. 10.1002/lio2.135 29492466 PMC5824112

[B19] Connor R. J. Kawaoka Y. Webster R. G. Paulson J. C. (1994). Receptor specificity in human, avian, and equine H2 and H3 influenza virus isolates. *Virology* 205 17–23. 10.1006/viro.1994.1615 7975212

[B20] Cooper K. W. Brann D. H. Farruggia M. C. Bhutani S. Pellegrino R. Tsukahara T. (2020). COVID-19 and the chemical Senses: Supporting players take center stage. *Neuron* 107 219–233. 10.1016/j.neuron.2020.06.032 32640192 PMC7328585

[B21] Croy I. Nordin S. Hummel T. (2014). Olfactory disorders and quality of life–an updated review. *Chem. Senses* 39 185–194. 10.1093/chemse/bjt072 24429163

[B22] Doty R. L. Mishra A. (2001). Olfaction and its alteration by nasal obstruction, rhinitis, and rhinosinusitis. *Laryngoscope* 111 409–423. 10.1097/00005537-200103000-00008 11224769 PMC7165948

[B23] Duncan H. J. Seiden A. M. (1995). Long-term follow-up of olfactory loss secondary to head trauma and upper respiratory tract infection. *Arch. Otolaryngol. Head Neck Surg.* 121 1183–1187. 10.1001/archotol.1995.01890100087015 7546588

[B24] Eisenberg R. J. Atanasiu D. Cairns T. M. Gallagher J. R. Krummenacher C. Cohen G. H. (2012). Herpes virus fusion and entry: A story with many characters. *Viruses* 4 800–832. 10.3390/v4050800 22754650 PMC3386629

[B25] Enwere E. Shingo T. Gregg C. Fujikawa H. Ohta S. Weiss S. (2004). Aging results in reduced epidermal growth factor receptor signaling, diminished olfactory neurogenesis, and deficits in fine olfactory discrimination. *J. Neurosci.* 24 8354–8365. 10.1523/JNEUROSCI.2751-04.2004 15385618 PMC6729689

[B26] Epstein V. A. Bryce P. J. Conley D. B. Kern R. C. Robinson A. M. (2008). Intranasal Aspergillus fumigatus exposure induces eosinophilic inflammation and olfactory sensory neuron cell death in mice. *Otolaryngol. Head Neck Surg.* 138 334–339. 10.1016/j.otohns.2007.11.029 18312881

[B27] Esiri M. M. (1982). Herpes simplex encephalitis. An immunohistological study of the distribution of viral antigen within the brain. *J. Neurol. Sci.* 54 209–226. 10.1016/0022-510X(82)90183-6 6284882

[B28] Féron F. Perry C. Mcgrath J. J. Mackay-Sim A. (1998). New techniques for biopsy and culture of human olfactory epithelial neurons. *Arch. Otolaryngol. Head Neck Surg.* 124 861–866. 10.1001/archotol.124.8.861 9708710

[B29] Fokkens W. J. Lund V. J. Hopkins C. Hellings P. W. Kern R. Reitsma S. (2020). European position paper on Rhinosinusitis and nasal polyps 2020. *Rhinology* 58 1–464. 10.4193/Rhin20.401 32077450

[B30] Genovese F. Tizzano M. (2018). Microvillous cells in the olfactory epithelium express elements of the solitary chemosensory cell transduction signaling cascade. *PLoS One* 13:e0202754. 10.1371/journal.pone.0202754 30212469 PMC6136699

[B31] Gevaert P. Calus L. Van Zele T. Blomme K. De Ruyck N. Bauters W. (2013). Omalizumab is effective in allergic and nonallergic patients with nasal polyps and asthma. *J Allergy Clin. Immunol.* 131:110–116.e111. 10.1016/j.jaci.2012.07.047 23021878

[B32] Glusman G. Yanai I. Rubin I. Lancet D. (2001). The complete human olfactory subgenome. *Genome Res.* 11 685–702. 10.1101/gr.171001 11337468

[B33] Godfrey P. A. Malnic B. Buck L. B. (2004). The mouse olfactory receptor gene family. *Proc. Natl. Acad. Sci. U.S.A.* 101 2156–2161. 10.1073/pnas.0308051100 14769939 PMC357068

[B34] Goncalves S. Goldstein B. J. (2016). Pathophysiology of olfactory disorders and potential treatment strategies. *Curr. Otorhinolaryngol. Rep.* 4 115–121. 10.1007/s40136-016-0113-5 27529054 PMC4982703

[B35] Graziadei P. P. Graziadei G. A. (1979). Neurogenesis and neuron regeneration in the olfactory system of mammals. I. Morphological aspects of differentiation and structural organization of the olfactory sensory neurons. *J. Neurocytol.* 8 1–18. 10.1007/BF01206454 438867

[B36] Gross E. A. Swenberg J. A. Fields S. Popp J. A. (1982). Comparative morphometry of the nasal cavity in rats and mice. *J. Anat.* 135 83–88. 7130058 PMC1168130

[B37] Gudis D. A. Soler Z. M. (2016). Chronic rhinosinusitis-related smell loss: Medical and surgical treatment efficacy. *Curr. Otorhinolaryngol. Rep.* 4 142–147. 10.1007/s40136-016-0114-4 29623247 PMC5882070

[B38] Håglin S. Berghard A. Bohm S. (2020). Increased retinoic acid catabolism in olfactory sensory neurons activates dormant tissue-specific stem cells and accelerates age-related metaplasia. *J. Neurosci.* 40 4116–4129. 10.1523/JNEUROSCI.2468-19.2020 32385093 PMC7244205

[B39] Hahn C. G. Han L. Y. Rawson N. E. Mirza N. Borgmann-Winter K. Lenox R. H. (2005). In vivo and in vitro neurogenesis in human olfactory epithelium. *J. Comp. Neurol.* 483 154–163. 10.1002/cne.20424 15678478

[B40] Hasegawa-Ishii S. Imamura F. Nagayama S. Murata M. Shimada A. (2020). Differential effects of nasal inflammation and odor deprivation on layer-specific degeneration of the mouse olfactory bulb. *eNeuro* 7:ENEURO.0403-19.2020. 10.1523/ENEURO.0403-19.2020 32220858 PMC7168263

[B41] Hasegawa-Ishii S. Shimada A. Imamura F. (2019). Neuroplastic changes in the olfactory bulb associated with nasal inflammation in mice. *J. Allergy Clin. Immunol.* 143:978–989.e973. 10.1016/j.jaci.2018.09.028 30315829

[B42] Hoffmann M. Kleine-Weber H. Schroeder S. Krüger N. Herrler T. Erichsen S. (2020). SARS-CoV-2 cell entry depends on ACE2 and TMPRSS2 and is blocked by a clinically proven protease inhibitor. *Cell* 181:271–280.e278. 10.1016/j.cell.2020.02.052 32142651 PMC7102627

[B43] Holbrook E. H. Leopold D. A. Schwob J. E. (2005). Abnormalities of axon growth in human olfactory mucosa. *Laryngoscope* 115 2144–2154. 10.1097/01.MLG.0000181493.83661.CE 16369158

[B44] Holbrook E. H. Wu E. Curry W. T. Lin D. T. Schwob J. E. (2011). Immunohistochemical characterization of human olfactory tissue. *Laryngoscope* 121 1687–1701. 10.1002/lary.21856 21792956 PMC3181071

[B45] Horowitz L. F. Saraiva L. R. Kuang D. Yoon K. H. Buck L. B. (2014). Olfactory receptor patterning in a higher primate. *J. Neurosci.* 34 12241–12252. 10.1523/JNEUROSCI.1779-14.2014 25209267 PMC4160765

[B46] Hummel T. Rothbauer C. Barz S. Grosser K. Pauli E. Kobal G. (1998a). Olfactory function in acute rhinitis. *Ann. N. Y. Acad. Sci.* 855 616–624. 10.1111/j.1749-6632.1998.tb10632.x 9929658

[B47] Hummel T. Rothbauer C. Pauli E. Kobal G. (1998b). Effects of the nasal decongestant oxymetazoline on human olfactory and intranasal trigeminal function in acute rhinitis. *Eur. J. Clin. Pharmacol.* 54 521–528. 10.1007/s002280050507 9832293

[B48] Igarashi K. M. Mori K. (2005). Spatial representation of hydrocarbon odorants in the ventrolateral zones of the rat olfactory bulb. *J. Neurophysiol.* 93 1007–1019. 10.1152/jn.00873.2004 15385587

[B49] Imai T. Sakano H. Vosshall L. B. (2010). Topographic mapping–the olfactory system. *Cold Spring Harb. Perspect. Biol.* 2:a001776. 10.1101/cshperspect.a001776 20554703 PMC2908763

[B50] Imamura F. Hasegawa-Ishii S. (2016). Environmental toxicants-induced immune responses in the olfactory mucosa. *Front. Immunol.* 7:475. 10.3389/fimmu.2016.00475 27867383 PMC5095454

[B51] Jafek B. W. (1983). Ultrastructure of human nasal mucosa. *Laryngoscope* 93 1576–1599. 10.1288/00005537-198312000-00011 6645759

[B52] Jafek B. W. Murrow B. Michaels R. Restrepo D. Linschoten M. (2002). Biopsies of human olfactory epithelium. *Chem. Senses* 27 623–628. 10.1093/chemse/27.7.623 12200342

[B53] Kalinke U. Bechmann I. Detje C. N. (2011). Host strategies against virus entry via the olfactory system. *Virulence* 2 367–370. 10.4161/viru.2.4.16138 21758005

[B54] Kanaya K. Kondo K. Suzukawa K. Sakamoto T. Kikuta S. Okada K. (2014). Innate immune responses and neuroepithelial degeneration and regeneration in the mouse olfactory mucosa induced by intranasal administration of Poly(I:C). *Cell Tissue Res.* 357 279–299. 10.1007/s00441-014-1848-2 24744264 PMC4077259

[B55] Kashiwagi T. Tsunemi Y. Akutsu M. Nakajima I. Haruna S. (2019). Postoperative evaluation of olfactory dysfunction in eosinophilic chronic rhinosinusitis - comparison of histopathological and clinical findings. *Acta Otolaryngol.* 139 881–889. 10.1080/00016489.2019.1654131 31438745

[B56] Kern R. C. (2000). Chronic sinusitis and anosmia: Pathologic changes in the olfactory mucosa. *Laryngoscope* 110 1071–1077. 10.1097/00005537-200007000-00001 10892672

[B57] Kikuta S. Han B. Yamasoba T. (2023). Heterogeneous damage to the olfactory epithelium in patients with post-viral olfactory dysfunction. *J. Clin. Med.* 12:5007. 10.3390/jcm12155007 37568409 PMC10419384

[B58] Kikuta S. Kuboki A. Yamasoba T. (2021). Protective effect of insulin in mouse nasal mucus against olfactory epithelium injury. *Front. Neural Circ.* 15:803769. 10.3389/fncir.2021.803769 35002636 PMC8733614

[B59] Kikuta S. Matsumoto Y. Kuboki A. Nakayama T. Asaka D. Otori N. (2016). Longer latency of sensory response to intravenous odor injection predicts olfactory neural disorder. *Sci. Rep.* 6:35361. 10.1038/srep35361 27734933 PMC5062120

[B60] Kikuta S. Sakamoto T. Nagayama S. Kanaya K. Kinoshita M. Kondo K. (2015). Sensory deprivation disrupts homeostatic regeneration of newly generated olfactory sensory neurons after injury in adult mice. *J. Neurosci.* 35 2657–2673. 10.1523/JNEUROSCI.2484-14.2015 25673857 PMC6605607

[B61] Kuboki A. Kikuta S. Otori N. Kojima H. Matsumoto I. Reisert J. (2021). Insulin-dependent maturation of newly generated olfactory sensory neurons after injury. *eNeuro* 8:ENEURO.0168-21.2021. 10.1523/ENEURO.0168-21.2021 33906971 PMC8143024

[B62] Lane A. P. Turner J. May L. Reed R. (2010). A genetic model of chronic rhinosinusitis-associated olfactory inflammation reveals reversible functional impairment and dramatic neuroepithelial reorganization. *J. Neurosci.* 30 2324–2329. 10.1523/JNEUROSCI.4507-09.2010 20147558 PMC2957830

[B63] Lechien J. R. Chiesa-Estomba C. M. De Siati D. R. Horoi M. Le Bon S. D. Rodriguez A. (2020). Olfactory and gustatory dysfunctions as a clinical presentation of mild-to-moderate forms of the coronavirus disease (COVID-19): A multicenter European study. *Eur. Arch. Otorhinolaryngol.* 277 2251–2261. 10.1007/s00405-020-05965-1 32253535 PMC7134551

[B64] Lemons K. Fu Z. Aoudé I. Ogura T. Sun J. Chang J. (2017). Lack of TRPM5-expressing microvillous cells in mouse main olfactory epithelium leads to impaired odor-evoked responses and olfactory-guided behavior in a challenging chemical environment. *eNeuro* 4:ENEURO.0135-17.2017. 10.1523/ENEURO.0135-17.2017 28612045 PMC5467397

[B65] Li L. Walker T. L. Zhang Y. Mackay E. W. Bartlett P. F. (2010). Endogenous interferon gamma directly regulates neural precursors in the non-inflammatory brain. *J. Neurosci.* 30 9038–9050. 10.1523/JNEUROSCI.5691-09.2010 20610738 PMC6632462

[B66] Liu Z. Y. Vaira L. A. Boscolo-Rizzo P. Walker A. Hopkins C. (2023). Post-viral olfactory loss and parosmia. *BMJ Med.* 2:e000382. 10.1136/bmjmed-2022-000382 37841969 PMC10568123

[B67] Malnic B. Godfrey P. A. Buck L. B. (2004). The human olfactory receptor gene family. *Proc. Natl. Acad. Sci. U.S.A.* 101 2584–2589. 10.1073/pnas.0307882100 14983052 PMC356993

[B68] Marin C. Hummel T. Liu Z. Mullol J. (2022). Chronic Rhinosinusitis and COVID-19. *J. Allergy Clin. Immunol. Pract.* 10 1423–1432. 10.1016/j.jaip.2022.03.003 35307579 PMC8926942

[B69] Meisami E. Mikhail L. Baim D. Bhatnagar K. P. (1998). Human olfactory bulb: Aging of glomeruli and mitral cells and a search for the accessory olfactory bulb. *Ann. N. Y. Acad. Sci.* 855 708–715. 10.1111/j.1749-6632.1998.tb10649.x 9929675

[B70] Moran D. T. Rowley J. C. III Jafek B. W. (1982). Electron microscopy of human olfactory epithelium reveals a new cell type: The microvillar cell. *Brain Res.* 253 39–46. 10.1016/0006-8993(82)90671-0 7150975

[B71] Mori I. Goshima F. Imai Y. Kohsaka S. Sugiyama T. Yoshida T. (2002). Olfactory receptor neurons prevent dissemination of neurovirulent influenza A virus into the brain by undergoing virus-induced apoptosis. *J. Gen. Virol.* 83 2109–2116. 10.1099/0022-1317-83-9-2109 12185263

[B72] Mori I. Nishiyama Y. Yokochi T. Kimura Y. (2004). Virus-induced neuronal apoptosis as pathological and protective responses of the host. *Rev. Med. Virol.* 14 209–216. 10.1002/rmv.426 15248249

[B73] Mori K. Sakano H. (2011). How is the olfactory map formed and interpreted in the mammalian brain? *Annu. Rev. Neurosci.* 34 467–499. 10.1146/annurev-neuro-112210-112917 21469960

[B74] Mori K. Sakano H. (2021). Olfactory circuitry and behavioral decisions. *Annu. Rev. Physiol.* 83 231–256. 10.1146/annurev-physiol-031820-092824 33228453

[B75] Morrison E. E. Costanzo R. M. (1990). Morphology of the human olfactory epithelium. *J. Comp. Neurol.* 297 1–13. 10.1002/cne.902970102 2376627

[B76] Nakashima T. Kimmelman C. P. Snow J. B. Jr. (1984). Structure of human fetal and adult olfactory neuroepithelium. *Arch. Otolaryngol.* 110 641–646. 10.1001/archotol.1984.00800360013003 6477257

[B77] Ogura T. Szebenyi S. A. Krosnowski K. Sathyanesan A. Jackson J. Lin W. (2011). Cholinergic microvillous cells in the mouse main olfactory epithelium and effect of acetylcholine on olfactory sensory neurons and supporting cells. *J. Neurophysiol.* 106 1274–1287. 10.1152/jn.00186.2011 21676931 PMC3174807

[B78] O’leary C. E. Schneider C. Locksley R. M. (2019). Tuft cells-systemically dispersed sensory epithelia integrating immune and neural circuitry. *Annu. Rev. Immunol.* 37 47–72. 10.1146/annurev-immunol-042718-041505 30379593 PMC8352721

[B79] Omura K. Han B. Nishijima H. Aoki S. Ebihara T. Kondo K. (2022). Heterogeneous distribution of mature olfactory sensory neurons in human olfactory epithelium. *Int. Forum Allergy Rhinol.* 12 266–277. 10.1002/alr.22885 34538025

[B80] Paik S. I. Lehman M. N. Seiden A. M. Duncan H. J. Smith D. V. (1992). Human olfactory biopsy. The influence of age and receptor distribution. *Arch. Otolaryngol. Head Neck Surg.* 118 731–738. 10.1001/archotol.1992.01880070061012 1627295

[B81] Perera-Lecoin M. Meertens L. Carnec X. Amara A. (2013). Flavivirus entry receptors: An update. *Viruses* 6 69–88. 10.3390/v6010069 24381034 PMC3917432

[B82] Rebholz H. Braun R. J. Ladage D. Knoll W. Kleber C. Hassel A. W. (2020). Loss of olfactory function-early indicator for Covid-19, other viral infections and neurodegenerative disorders. *Front. Neurol.* 11:569333. 10.3389/fneur.2020.569333 33193009 PMC7649754

[B83] Ressler K. J. Sullivan S. L. Buck L. B. (1993). A zonal organization of odorant receptor gene expression in the olfactory epithelium. *Cell* 73 597–609. 10.1016/0092-8674(93)90145-G 7683976

[B84] Richard M. B. Taylor S. R. Greer C. A. (2010). Age-induced disruption of selective olfactory bulb synaptic circuits. *Proc. Natl. Acad. Sci. U.S.A.* 107 15613–15618. 10.1073/pnas.1007931107 20679234 PMC2932573

[B85] Rombaux P. Huart C. Levie P. Cingi C. Hummel T. (2016). Olfaction in Chronic rhinosinusitis. *Curr. Allergy Asthma Rep.* 16:41. 10.1007/s11882-016-0617-6 27131498

[B86] Rosenfeld R. M. Piccirillo J. F. Chandrasekhar S. S. Brook I. Ashok Kumar K. Kramper M. (2015). Clinical practice guideline (update): Adult sinusitis. *Otolaryngol. Head Neck Surg.* 152 S1–S39. 10.1177/0194599815572097 25832968

[B87] Rudmik L. Hoy M. Schlosser R. J. Harvey R. J. Welch K. C. Lund V. (2013). Topical therapies in the management of chronic rhinosinusitis: An evidence-based review with recommendations. *Int. Forum Allergy Rhinol.* 3 281–298. 10.1002/alr.21096 23044832

[B88] Saunders C. J. Christensen M. Finger T. E. Tizzano M. (2014). Cholinergic neurotransmission links solitary chemosensory cells to nasal inflammation. *Proc. Natl. Acad. Sci. U.S.A.* 111 6075–6080. 10.1073/pnas.1402251111 24711432 PMC4000837

[B89] Schlosser R. J. Mulligan J. K. Hyer J. M. Karnezis T. T. Gudis D. A. Soler Z. M. (2016). Mucous cytokine levels in chronic rhinosinusitis-associated olfactory loss. *JAMA Otolaryngol. Head Neck Surg.* 142 731–737. 10.1001/jamaoto.2016.0927 27228459 PMC5751717

[B90] Seiden A. M. (2004). Postviral olfactory loss. *Otolaryngol. Clin. North Am.* 37 1159–1166. 10.1016/j.otc.2004.06.007 15563908

[B91] Selvaraj S. Liu K. Robinson A. M. Epstein V. A. Conley D. B. Kern R. C. (2012). In vivo determination of mouse olfactory mucus cation concentrations in normal and inflammatory states. *PLoS One* 7:e39600. 10.1371/journal.pone.0039600 22911687 PMC3401282

[B92] Soler Z. M. Sauer D. A. Mace J. Smith T. L. (2009). Relationship between clinical measures and histopathologic findings in chronic rhinosinusitis. *Otolaryngol. Head Neck Surg.* 141 454–461. 10.1016/j.otohns.2009.06.085 19786212 PMC2766519

[B93] Sui Y. Horne M. K. Stanić D. (2012). Reduced proliferation in the adult mouse subventricular zone increases survival of olfactory bulb interneurons. *PLoS One* 7:e31549. 10.1371/journal.pone.0031549 22363671 PMC3283653

[B94] Takahashi Y. K. Kurosaki M. Hirono S. Mori K. (2004). Topographic representation of odorant molecular features in the rat olfactory bulb. *J. Neurophysiol.* 92 2413–2427. 10.1152/jn.00236.2004 15152015

[B95] Torabi A. Mohammadbagheri E. Akbari Dilmaghani N. Bayat A. H. Fathi M. Vakili K. (2020). Proinflammatory cytokines in the olfactory mucosa result in COVID-19 induced anosmia. *ACS Chem. Neurosci.* 11 1909–1913. 10.1021/acschemneuro.0c00249 32525657

[B96] Ualiyeva S. Hallen N. Kanaoka Y. Ledderose C. Matsumoto I. Junger W. G. (2020). Airway brush cells generate cysteinyl leukotrienes through the ATP sensor P2Y2. *Sci. Immunol.* 5:eaax7224. 10.1126/sciimmunol.aax7224 31953256 PMC7176051

[B97] Van Riel D. Leijten L. M. Verdijk R. M. Geurtsvankessel C. Van Der Vries E. Van Rossum A. M. (2014). Evidence for influenza virus CNS invasion along the olfactory route in an immunocompromised infant. *J. Infect Dis.* 210 419–423. 10.1093/infdis/jiu097 24550441

[B98] Van Riel D. Verdijk R. Kuiken T. (2015). The olfactory nerve: A shortcut for influenza and other viral diseases into the central nervous system. *J. Pathol.* 235 277–287. 10.1002/path.4461 25294743

[B99] Victores A. J. Chen M. Smith A. Lane A. P. (2018). Olfactory loss in chronic rhinosinusitis is associated with neuronal activation of c-Jun N-terminal kinase. *Int. Forum Allergy Rhinol.* 8 415–420. 10.1002/alr.22053 29193850 PMC5842118

[B100] Villar E. Barroso I. M. (2006). Role of sialic acid-containing molecules in paramyxovirus entry into the host cell: A minireview. *Glycoconj. J.* 23 5–17. 10.1007/s10719-006-5433-0 16575518

[B101] Vogalis F. Hegg C. C. Lucero M. T. (2005). Ionic conductances in sustentacular cells of the mouse olfactory epithelium. *J. Physiol.* 562 785–799. 10.1113/jphysiol.2004.079228 15611020 PMC1665525

[B102] Welge-Lüssen A. Wolfensberger M. (2006). Olfactory disorders following upper respiratory tract infections. *Adv. Otorhinolaryngol.* 63 125–132. 10.1159/000093758 16733337

[B103] Wrobel B. B. Leopold D. A. (2004). Clinical assessment of patients with smell and taste disorders. *Otolaryngol. Clin. North Am.* 37 1127–1142. 10.1016/j.otc.2004.06.010 15563906 PMC7118991

[B104] Wu D. Li Y. Bleier B. S. Wei Y. (2020). Superior turbinate eosinophilia predicts olfactory decline in patients with chronic rhinosinusitis. *Ann. Allergy Asthma Immunol.* 125:304–310.e301. 10.1016/j.anai.2020.04.027 32387168

[B105] Yamada M. Nakamura K. Yoshii M. Kaku Y. Narita M. (2009). Brain lesions induced by experimental intranasal infection of Japanese encephalitis virus in piglets. *J. Comp. Pathol.* 141 156–162. 10.1016/j.jcpa.2009.04.006 19523649

[B106] Yan X. Whitcroft K. L. Hummel T. (2020). Olfaction: Sensitive indicator of inflammatory burden in chronic rhinosinusitis. *Laryngosc. Invest. Otolaryngol.* 5 992–1002. 10.1002/lio2.485 33364387 PMC7752087

[B107] Yan C. H. Faraji F. Prajapati D. P. Boone C. E. Deconde A. S. (2020). Association of chemosensory dysfunction and COVID-19 in patients presenting with influenza-like symptoms. *Int. Forum Allergy Rhinol.* 10 806–813. 10.1002/alr.22579 32279441 PMC7262089

[B108] Yee K. K. Pribitkin E. A. Cowart B. J. Rosen D. Feng P. Rawson N. E. (2009). Analysis of the olfactory mucosa in chronic rhinosinusitis. *Ann. N. Y. Acad. Sci.* 1170 590–595. 10.1111/j.1749-6632.2009.04364.x 19686198 PMC2729508

[B109] Yee K. K. Pribitkin E. A. Cowart B. J. Vainius A. A. Klock C. T. Rosen D. (2010). Neuropathology of the olfactory mucosa in chronic rhinosinusitis. *Am. J. Rhinol. Allergy* 24 110–120. 10.2500/ajra.2010.24.3435 20021743 PMC5903554

[B110] Young J. M. Friedman C. Williams E. M. Ross J. A. Tonnes-Priddy L. Trask B. J. (2002). Different evolutionary processes shaped the mouse and human olfactory receptor gene families. *Hum. Mol. Genet.* 11 535–546. 10.1093/hmg/11.5.535 11875048

[B111] Youngentob S. L. Schwob J. E. Sheehe P. R. Youngentob L. M. (1997). Odorant threshold following methyl bromide-induced lesions of the olfactory epithelium. *Physiol. Behav.* 62 1241–1252. 10.1016/S0031-9384(97)00301-6 9383109

[B112] Yousem D. M. Geckle R. J. Bilker W. B. Doty R. L. (1998). Olfactory bulb and tract and temporal lobe volumes. Normative data across decades. *Ann. N. Y. Acad. Sci.* 855 546–555. 10.1111/j.1749-6632.1998.tb10624.x 9929650

[B113] Zhang X. Firestein S. (2002). The olfactory receptor gene superfamily of the mouse. *Nat. Neurosci.* 5 124–133. 10.1038/nn800 11802173

[B114] Zhao K. Jiang J. Pribitkin E. A. Dalton P. Rosen D. Lyman B. (2014). Conductive olfactory losses in chronic rhinosinusitis? A computational fluid dynamics study of 29 patients. *Int. Forum Allergy Rhinol.* 4 298–308. 10.1002/alr.21272 24449655 PMC4144185

[B115] Zozulya S. Echeverri F. Nguyen T. (2001). The human olfactory receptor repertoire. *Genome Biol.* 2:Research0018. 10.1186/gb-2001-2-6-research0018 11423007 PMC33394

